# Strategies used by international sports federations in the field of technology

**DOI:** 10.3389/fspor.2026.1726682

**Published:** 2026-07-22

**Authors:** Michel Desbordes, Thomas Rywalski

**Affiliations:** Institute of Sport Sciences (ISSUL), University of Lausanne, Lausanne, Switzerland

**Keywords:** digital transformation, fan engagement, fan experience, international sports federations, new technologies, sport innovation

## Abstract

This research explores how international sports federations (IFs) use new technologies to enhance at their events. It overviews the digital initiatives and tools deployed to improve in-person and remote experiences. The study analyses two main questions: which technologies IFs use most often and how they implement them to improve international sports events’ quality and attractiveness. The researcher applied a mixed-methods approach by analysing 115 initiatives from Summer Olympic IFs and 65 from Winter Olympic IFs and conducting qualitative interviews with 11 IFs representatives and one academic expert. The findings show cautious optimism about Virtual Reality (VR), Augmented Reality (AR), and Artificial Intelligence (AI) for enhancing fan experiences. IFs mainly use AR for broadcast and advertising, though technical and financial constraints limit its application. VR offers immersive potential but remains niche and costly. However, AI is more widely adopted for automation and content generation but faces realism challenges. The second observation is that customer relationship management systems also play a key role in enabling federations to tailor content and services to fans’ preferences. Furthermore, many IFs use data analytics to monitor fan behaviour and improve engagement through digital platforms like websites, mobile apps, and OTT services. The study also highlights that federations centralising fan data from multiple sources demonstrate higher personalised experiences. Despite financial and organisational constraints, several federations manage to implement impactful technologies with limited resources. Thus, this exploratory research brings new perspectives to the field of sport management by identifying current practices, suggesting practical recommendations and providing the basis for future studies on organisational intentions and practices about digital innovation in international sport.

## 1 Introduction

“The global sports industry is professionalizing, with heightened stakes and increasing investments in talent, analytics, and fan experiences” (1).

The days when sports fans went to the stadium to watch the game and left straight away may be over. The trend is moving towards a more exciting matchday experience. Thus, fans arrive early to take advantage of the entertainment zone inside the stadium complex for food, shopping, and entertainment. No matter the match's result, they celebrate in the stadium bars. These moments of sharing between fans create a fun and memorable moment for them.

Therefore, by owning and managing mixed-use entertainment areas, the organisers of today's major sporting events are generating new sources of income and at the same time enhancing the fan experience. These zones typically include shops, restaurants, entertainment venues, offices and living spaces. As a result, visitors enjoy themselves all year round, strengthening the link between the sports organisation and the spectators.

Each of the five major sports leagues has submitted new venue plans for projects that include entertainment areas throughout the last two years (2). According to PWC (2), every significant new venue development project announced in 2024 will centre around entertainment areas and, consequently, fan experience.

This article is organised into five main sections.^1^ Firstly, it begins with the Introduction, which outlines the background and aims of the study. It brings contextual information such as the evolution of fan experiences beyond the game day and the growing role of artificial intelligence in sport. Then, it is followed by the Materials and Methods. This part focuses on the field of study and explains the methodology used in the research. It combines descriptive analysis and qualitative methods to gather and analyse data. It also presents the interview profiles and outlines how primary and secondary data were collected. Then, the Results analysis section is introduced with a structuration into two themes corresponding to the research questions. Following the results, the Discussion reviews the three hypotheses and analyses the findings about existing knowledge. The conclusion ultimately takes a look at the matter from a broader perspective in order to present the subject in a wider context.

- First research's question: What are the key technologies improving fan experience in international sports events?
- Second research's question: How International Sports Federations use new technologies to improve user experience at their sports events?

Considering the methodology we used in the following section, the reader will see that our article can be considered more as an “exploratory research” than a real strict robust hypothesis validation.

## 2 Materials and methods

### 2.1 Research's questions

Q1. What are the key technologies improving used in international sports events?

Q2. How International Sports Federations use new technologies that could improve user experience at their sports events?

### 2.2 International sports events environment

#### 2.2.1 International sports events

Organising an international sports event presents unique challenges with broad implications for stakeholders. Thus, we must first agree on a definition. Inspired by Getz & Page (3), a general event comprises three characteristics. They define it as occurring at a specific time and place, shaped by certain conditions and recognised as a significant experience with a clear beginning and end.

More specifically, sports events can be described in the following way, as presented during the 31st International Seminar on Olympic Studies for Postgraduate Students (27). Above all, they are occasions “where men and women gather […] to watch a sport show” (4), para. 4; 31: p.23). In this context, sports events refer to the games or meetings during which sports activity occurs (3). Furthermore, they come in various formats, and we will focus on one specifically: an international event in scope (32). Moreover, different classifications exist in literature trying to group sports events as objectively as possible. As a matter of fact in the article “Major international sporting events”, a definition of international sports event was given by grouping the criteria of some authors (28–30, 34): “an international scope; a considerable number of participants, spectators and volunteers; appeal and extensive media coverage; high organisational expenses; significant impacts on the public and the built environment” (4). Given this context, the spectator of the international event becomes a point of particular interest.

#### 2.2.2 Sport experience design, fan experience and engagement

“The concept of sport experience design (SX design) represents the process of enhancing customer engagement by improving the use and pleasure provided in interactions between the customer and the delivery of the sport product or service” (5), p. 4). Then, the authors have defined three factors to describe the SX design better:

– The sport user
– The sport context
– The sport management context

The authors (5), p. 4) define the concepts: “The sport consumer has psychological tendencies related to perceptions, personalities, desires and emotions, as well as personal characteristics related to demographics. […] The sport context is the physical and virtual environments in which the specific sport behaviour takes place. […] The sport management system is the process through which a sport organisation creates and delivers the sport product or service to the customer”.

#### 2.2.3 Sport consumer behaviour

**Figure 1:**
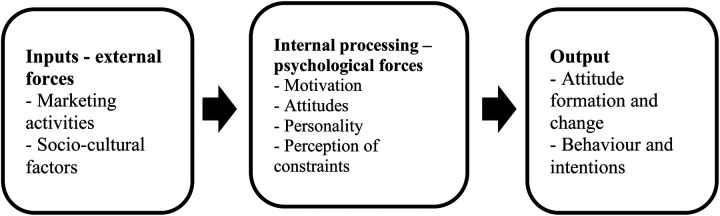
Sport consumer decision-making sequence. Reproduced from (5), sport consumer behaviour: marketing strategies).

As a key factor of the sport experience design (5), the authors, raise the need to study sport consumer behaviour as an experience from the consumer's point of view. Thus, the sports experience reflects the interactions between customers and providers of sports products or services, including enjoying these exchanges. They define sport consumer behaviour as the psychological and physical reactions occurring before, during, and after consuming a sport product or service. Physical responses can include physiological reactions of excitation and tension related to the sports experience, while psychological responses can include perceptions, feelings, and evaluations related to the sports experience. These mental and physical responses may manifest when looking for and choosing sports services (6). Participating in sports experiences that fulfil needs and desires and offer benefits is an essential concept of sport consumer behaviour. This explanation aids in the understanding of why people choose to invest their time, money, and effort in sporting activities (5). Figure 1 describes the sport consumer decision-making sequence in three stages.

### 2.3 Use of new technologies in international sports

The literature review reveals a noticeable lack of academic studies focusing on new technologies at sports events. Nevertheless, valuable insights on this topic are available within the “grey literature”, as presented below.

#### 2.3.1 Technology-driven transformation of fan experience

“Without fans, we don’t have professional sports” (Sports Tech HQ, 2024).

This quote underscores the increasing importance of innovative technologies in shaping how sports organisations engage with fans. The global sports technology ecosystem has experienced a significant shift in investment priorities, with a growing emphasis on fan solutions. The 2023 Global SportsTech Ecosystem Report highlighted that the “Fans & Content” segment attracted the most investment between 2018 and 2022, particularly in the APAC region (71.8%), followed by North America (46.2%) and Europe (41.7%) (7). This trend continued into 2024, with the USA the top destination for sports tech funding and Germany overtaking China as the second-highest recipient (8).

##### 2.3.1.1 Fan data: from fragmentation to integration

“Organizations need to understand how a technology meets fans’ expectations, what the ROI potential is, and whether services are sustainable” (9).

For sports organisations, fan data is increasingly central to strategy. As Capgemini (9) emphasises, fans expect technologies to deliver intuitive experiences, particularly when engaging with tools like VR streaming or stadium connectivity. Thus, when done well, these improve satisfaction and engagement.

Furthermore, transitioning from fragmented data to centralised fan profiles is a crucial step forward. Deloitte (10) illustrates how Major League Baseball and the NFL use integrated data to offer tailored promotions and loyalty rewards. According to their study, 70% of consumers are likelier to engage with personalised brand interactions. Similarly, Customer Data Platforms enable deeper insights by merging data from multiple sources (11).

Capgemini (12) further notes that consolidated data systems allow International Federations (IFs) to personalise content, deliver targeted offers, and enhance real-time interactions during events. By connecting data from broadcasters, organisers, and sponsors, IFs gain a competitive edge.

**Figure 2:**
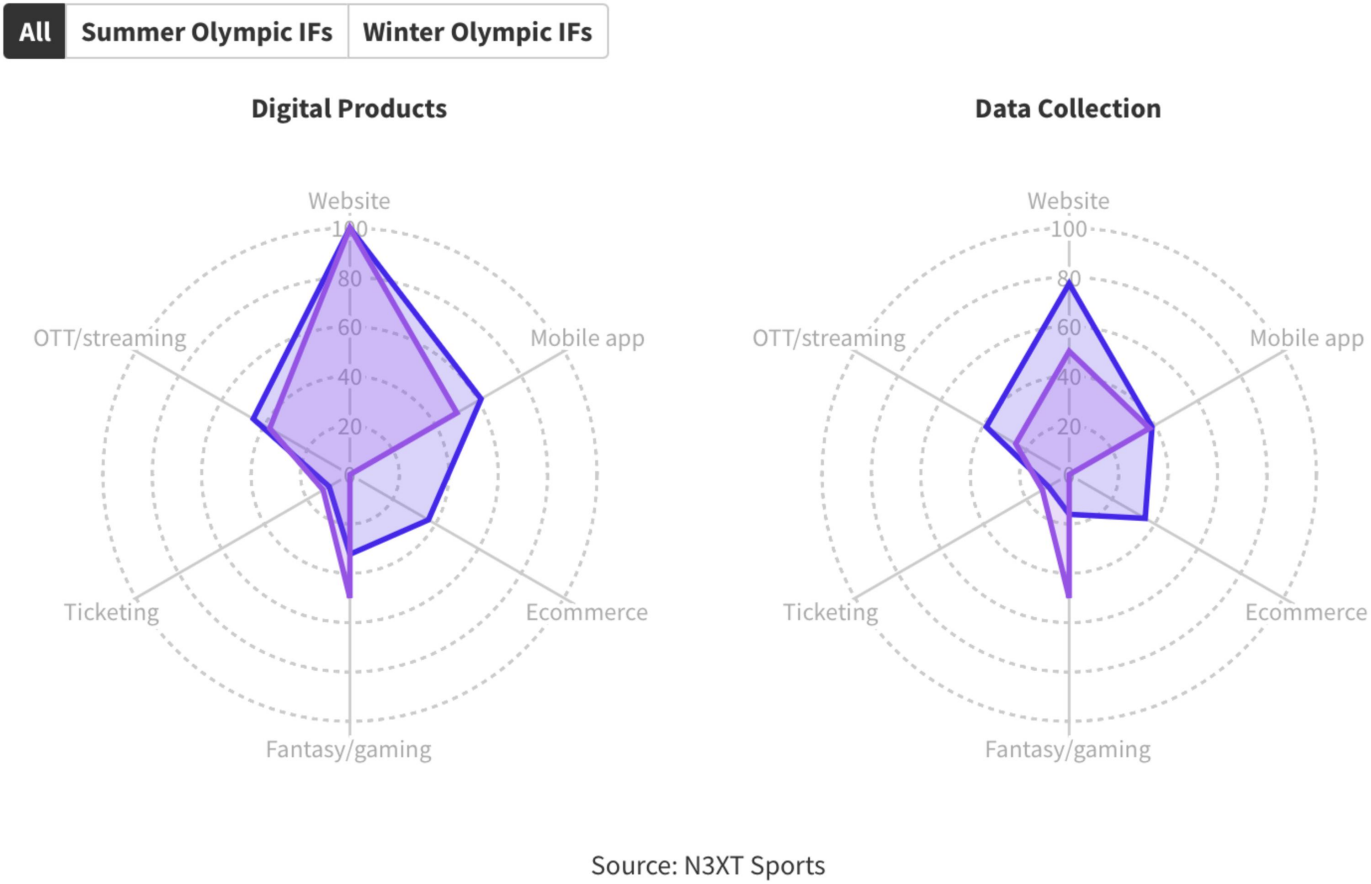
Summer vs. Winter IFs: digital & data analysis. Summer olympic IFs are represented in blue and Winter Olympic IFs in purple. Reprinted from (11).

Moreover, recent benchmarking of Olympic IFs shows that 75% of Winter IFs collect fan data, with 62.5% using diverse tools such as apps, newsletters, and prediction games. By contrast, only 50% of Summer IFs employ multichannel strategies (11). Thus, te IIHF Fan Zone and IBU's Single Sign-On platform exemplify mature digital ecosystems, while other IFs like the IBSF lag, concentrating more on athlete data than fan interaction. Figure 2 compares Summer and Winter International Federations for Digital & Data analysis.

Despite these advancements, many IFs are still early in their digital transformation. The International Federation Fan Data Guide 2024 notes a lack of comprehensive systems, especially in first-party data collection (13). During the 2022 IF Seminar, Ivan Khodabakhsh (Swiss Timing) stressed the need for centralised systems, noting that fragmented data held by LOCs, broadcasters, and federations hinders strategic insights. Furthermore, he identified digital maturity as a key enabler of effective fan engagement and organisational innovation (14).

#### 2.3.2 Enhancing the fan experience through innovation

“Personalized sports content is the ultimate end. The fans decide how they want to consume their sport of choice” (Sports Tech HQ, 2024).

**Figure 3:**
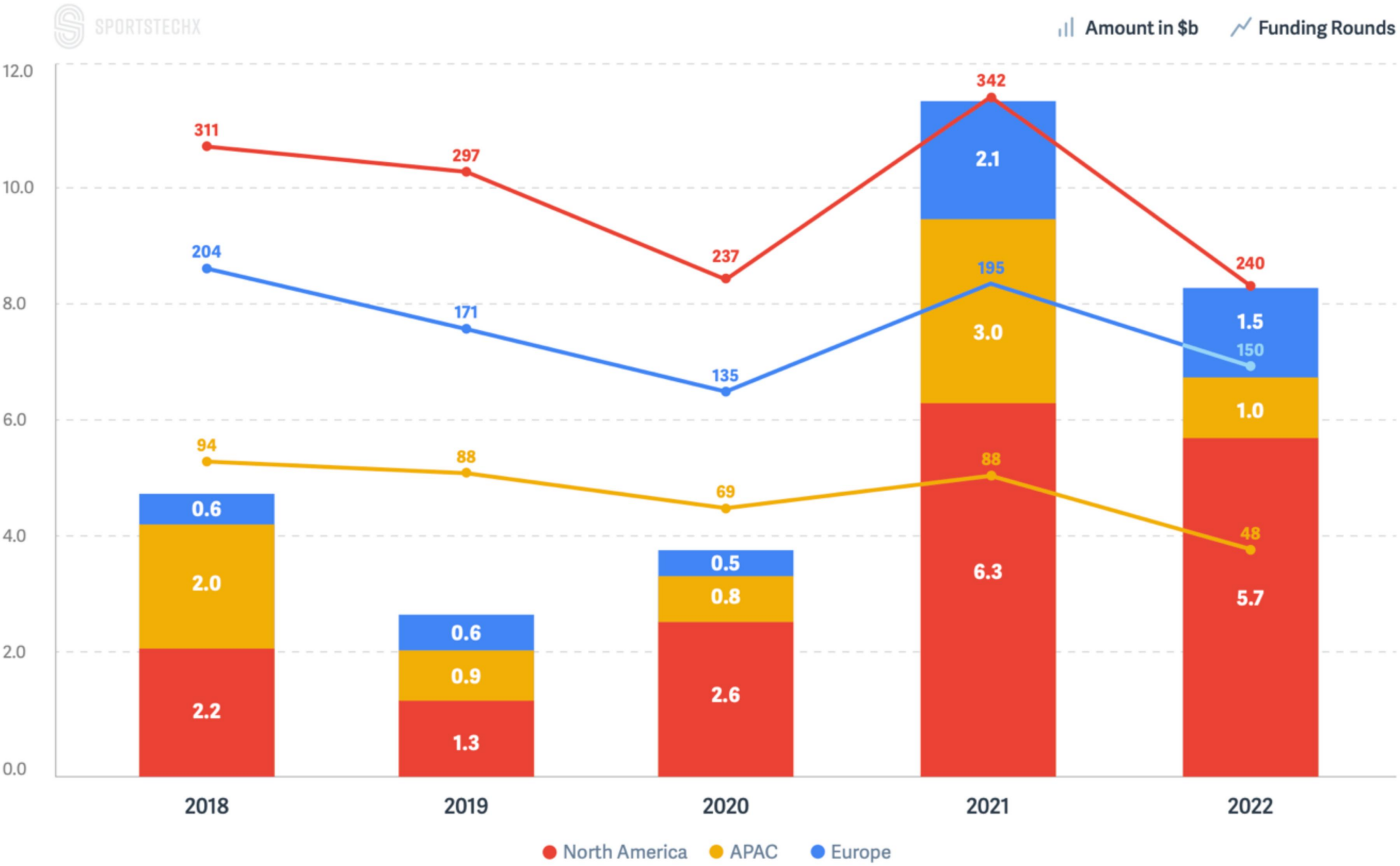
Global sportsTech funding by region (2018–2022). Reprinted from (7).

The global market reflects this trend. In 2022, 47% of all North American sports tech funding targeted fan experience, with Web3 and streaming projects receiving significant investments (7). In 2023, APAC allocated 73.7% of its funding to fan engagement, while Europe and North America showed smaller but steady interest (8). Figure 3 analyses how SportsTech is funded in different parts of the globe.

As a result, technologies such as augmented reality, virtual reality, mobile apps and digital collectables are now the basis for fan engagement strategies. According to Capgemini (9), fans who value these technologies are likelier to spend money on merchandise and subscriptions. However, sports organisations must address data privacy concerns through transparent policies and incentive schemes for data sharing.

Accordingly, N3XT Sports (13) stresses that CRM systems are essential for understanding fan journeys and improving D2C strategies. These tools help identify audience segments and customise content at every touchpoint. In parallel, Teamworks and SportTechX (15) report that transforming casual fans into avid fans through hyper-personalised experiences enhances loyalty and spending. Their findings suggest that innovation should follow a step-by-step approach aligned with sports' seasonal character.

Moreover, only 50% of Olympic IFs allow digital subscribers to personalise their experience despite CRM availability, which is more than the FIFA World Cup (34%) or Rugby World Cup (35%) (13). Following this idea, EPFL's work at events like the Freeride World Tour and Youth Olympic Games shows how immersive apps and health-integrated digital experiences enhance engagement and wellbeing (16).

#### 2.3.3 The future of Fan engagement

Reports like State of Sports Fan Engagement 2023 (17) show that 71% of sports organisations consider fan engagement essential, with revenue generation as the top objective. Key channels retain:

- Social media (37%),
- OTT streaming (18%), and
- Mobile apps (13%).

**Figure 4:**
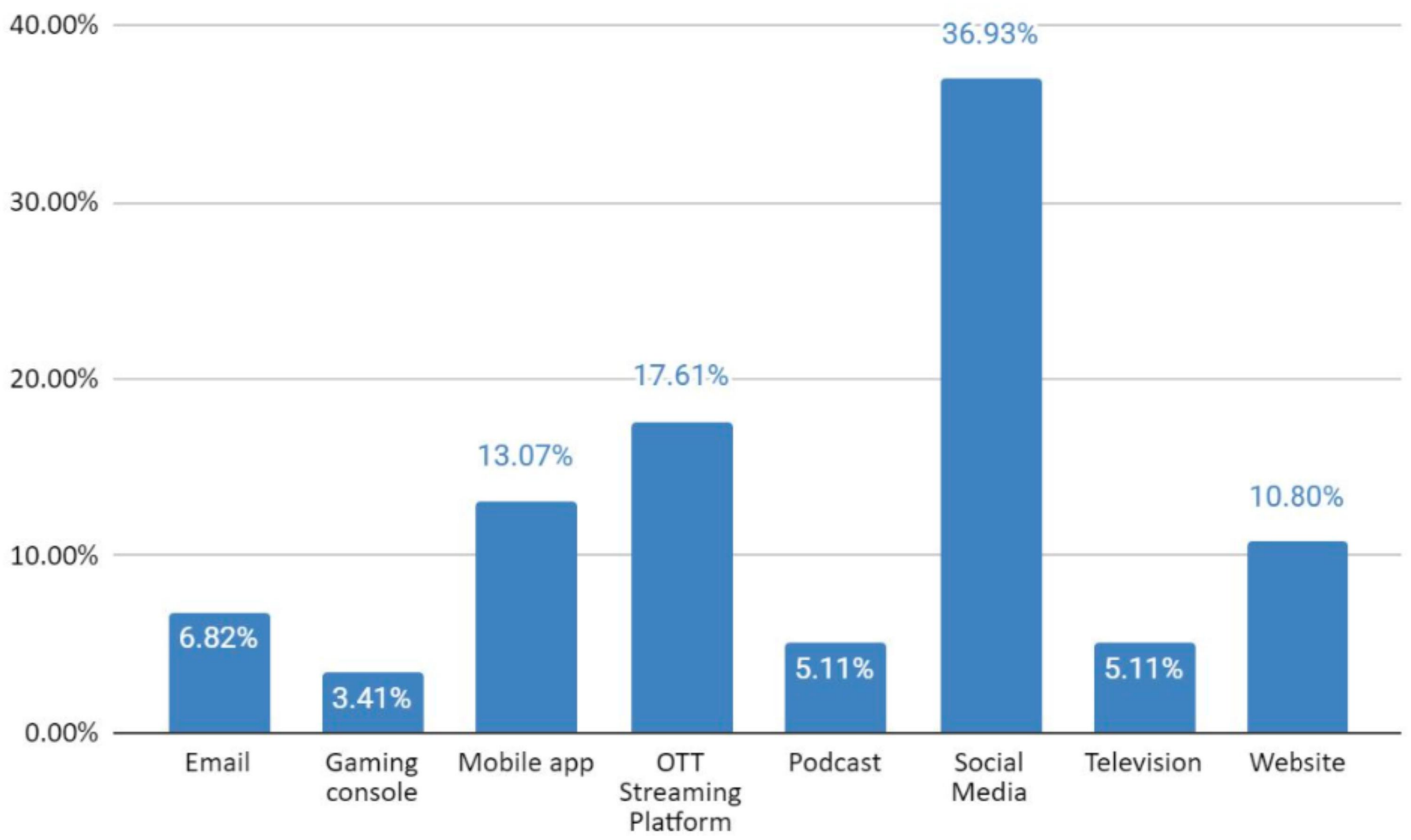
What do you consider to be the most important digital channels to enable your organisation to achieve its fan engagement goals? Reprinted from (17).

Mobile apps now serve as essential tools for delivering live scores, stats, streaming, and exclusive content, ensuring fans remain connected regardless of location (17, 18). Figure 4 presents the results of a survey conducted on digital channels in numerous organisations.

**Figure 5:**
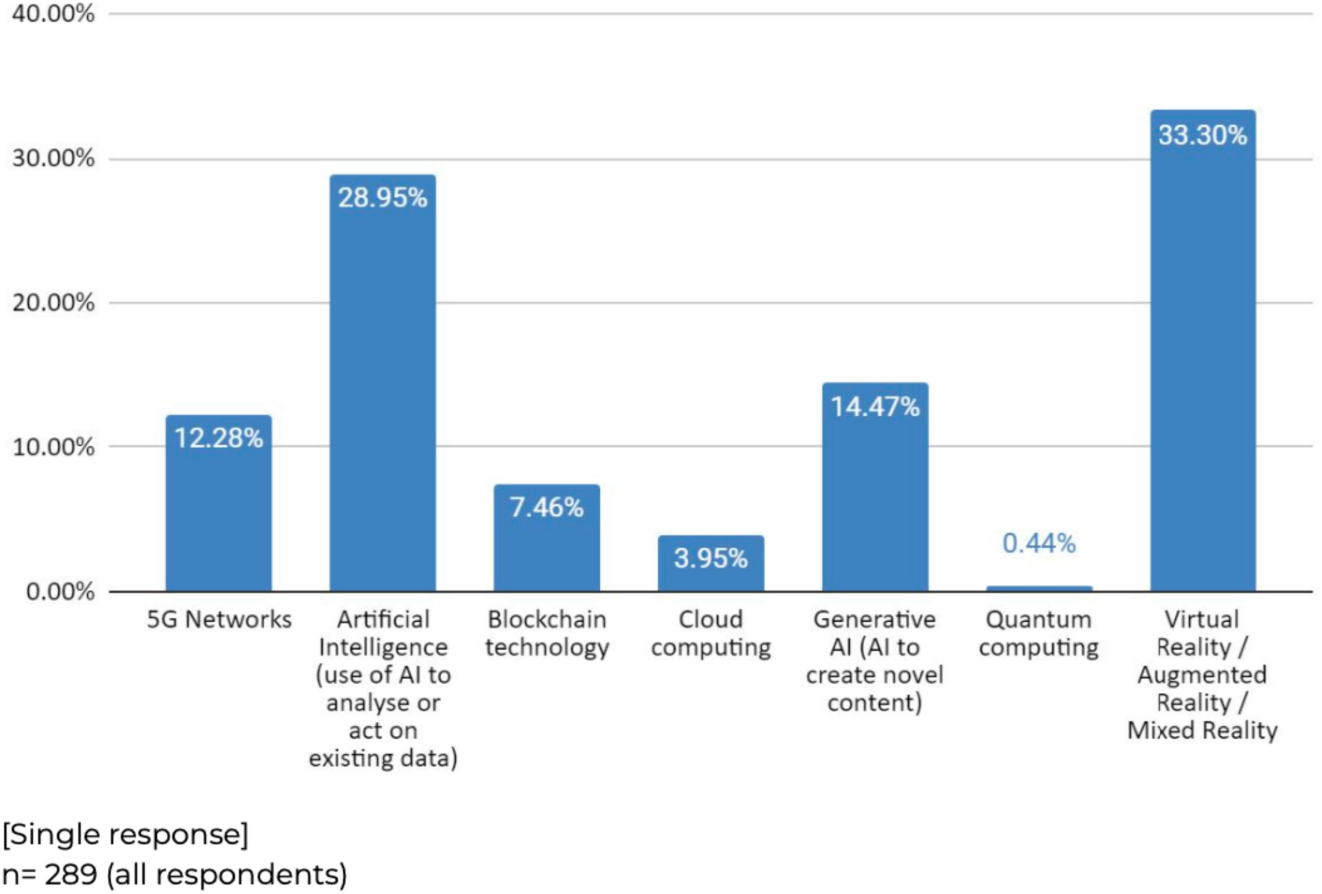
What single technology will be the most revolutionary for the field of fan engagement in the next 3–5 years? Reprinted from (17).

More specifically, AR enhances live viewing with real-time data overlays, while VR offers immersive virtual stadium experiences. Despite high costs, these tools significantly enrich fan interaction. Thus, sports organisations are shaping more fan engagement through personalised, multi-platform strategies. Figure 5 tries to show what are the predictions for the technologies considered as the “most revolutionary ones” in the field of fan engagement.

Furthermore, Artificial Intelligence (AI) plays a key role in driving this transformation. Recognised as the “next frontier” in fan engagement (10, 11), AI enables personalised experiences through chatbots, targeted emails, and customised video content. For instance, 60% of NBA teams use AI chatbots to assist fans with tickets, travel, and merchandise (13).

### 2.4 Presentation of hypotheses

#### 2.4.1 Hypothesis 1

*Virtual Reality (VR), Augmented Reality (AR), and Artificial Intelligence (AI) are the most frequently used technologies to enhance the fan experience at international sports events, and their implementation is profitable and sustainable in the long term.*

#### 2.4.2 Hypothesis 2

*Artificial Intelligence (AI) and Customer Relationship Management (CRM) systems enhance significantly fan experience through personalisation.*

#### 2.4.3 Hypothesis 3

*International Federations that consolidate fan data from various sources achieve higher digital maturity and improve fan engagement than those with fragmented data systems.*

This research adopts a mixed-methods approach, combining both secondary and primary data collection to provide a comprehensive understanding of the use of new technologies by International Federations (IFs). Secondary data offers an overall, objective overview of trends in technology use, while primary data, gathered through interviews, provides in-depth insights from experts in the field.

### 2.5 Framework

#### 2.5.1 Secondary data

The first phase of the research framework concerns collecting secondary data from various sources. To do so, I looked for official IF websites, annual reports, consultancy studies, and academic publications. This dataset is then analysed to identify key trends in adopting and implementing technologies within IFs. To facilitate analysis, I created structured tables summarising the findings. It provides the foundational context for the research and informs the primary data collection method.

#### 2.5.2 Primary data

The second data collection phase focuses on primary data following the secondary data analysis. It includes both qualitative and descriptive analysis. I compile qualitative data through semi-structured interviews with key stakeholders in IFs, while I conduct descriptive analysis from the structured tables created from the secondary data. Integrating both data types allowed me to understand how technology is implemented and its impact.

### 2.6 Descriptive analysis research

The primary aim of the descriptive analysis is to describe and categorise the different technological initiatives implemented by IFs. The first step in the data collection process is to extract relevant information from official sources and industry reports. I then categorised these initiatives by technology type (e.g., AI, AR, VR, CRM, Streaming,…) and used GenAI to identify their purposes. These different steps allowed to create a comprehensive database that facilitated analysis. We visualised this dataset through graphs and tables to compare trends across federations. We also compared technological initiatives between Winter and Summer IFs to identify specific patterns.

### 2.7 Qualitative research

For the interviews, we aimed to ensure a representative selection of interviewees from both the ASOIF and AIOWF International Federation groups. We intentionally tried to represent each group by selecting individuals from IFs. To achieve this, we reviewed the roles of employees in each federation. We identified those whose work is most closely related to fan engagement or experience, even when fan experience may not always be part of their official titles. We focused on roles linked to digital marketing, IT, and commercial activities. We successfully conducted interviews based on the contacts established and their responses.

After interviewing, we compiled a table of interviewee profiles to contextualise the interview findings, including their roles and affiliations. Then, we transcribed the interviews and read them multiple times to identify emerging themes. After that, these themes were categorised into a structured table, allowing a precise analysis. The qualitative data were imported into NVivo to facilitate the coding process and thematic analysis (Nvivo 14 Release 14.24.0).

Consequently, the combination of qualitative and descriptive analysis enhances the reliability of the findings, as the qualitative interviews can confirm or challenge the insights gained from the descriptive analysis. Table 1 presents the list of experts that were interviewed to answer the research questions.

**Table 1:** Table of interviewees.

Interview number	Name	IF	Job position	Interview date	Duration	Methods
1	Gaspard Dufour	FEI	Director of Technology and Sports Services	21.11.2024	00:47:12	In person
2	Bernardo Franco	Volleyball World	Head of Technology	27.11.2024	00:34:42	In person
3	Stéphane Schwander	FEI	Head of Content and Channel Management	02.12.2024	00:46:56	In person
4	Harsimran Singh Virk	FIH	Senior Technology & Digital Manager	02.12.2024	00:33:07	In person
5	Hilary Atkinson	FIH	FIH Pro League and Olympic Games Director	02.12.2024	00:13:03	In person
6	Jon Wyatt	FIH	Interim Senior Director	02.12.2024	00:20:12	In person
7	Adrien De Cheveigné	UCI	Head of Digital Transformation and Strategic Development	04.12.2024	00:50:20	In person
8	Ian Troseder	ISU	Marketing Manager - (digital/data)	11.12.2024	00:34:33	In person
9	Cyril Nivel	ICF	Global Director of Operations	11.12.2024	00:48:56	In person
10	Paul O’Neil	FIG	Head of Communications	20.12.2024	00:44:43	In person
11	Ekaterina Glebova	Academic expert	Professor in Sports Marketing	23.12.2024	00:00:00	By email

At this stage, we can start discuss the quality of our sample. 11 interviews can be considered as relatively strong, but it is obvious that this is not totally representative of the various field of the federations. According to Eisenhardt (19), a case study methodology would have been more adapted: therefore we would have needed to conduct four interviews per federation, and probably extend our research to a minimum of four federations. That's is why the following results should be considered as exploratory.

## 3 Results

The results section presents the findings of this study to answer research questions. The aim is to determine how International Federations (IFs) integrate new technology to enhance fans' experiences at their sporting events. The results are presented formally, starting with the descriptive analysis of IFs technology initiatives and then moving to qualitative information gained through interviews.

### 3.1 Q1. What are the key technologies improving used in international sports events?

#### 3.1.1 Overview of technology adoption across IFs

This section presents a descriptive analysis of results and identifies technological initiatives implemented by Summer and Winter IFs. The research analyses a sample of 116 Summer IFs initiatives and 65 Winter IFs initiatives, classifying them according to their primary and secondary technology types. It shows the technologies implemented most frequently, their distribution among federations, and the developed technological trends.

**Figure 6:**
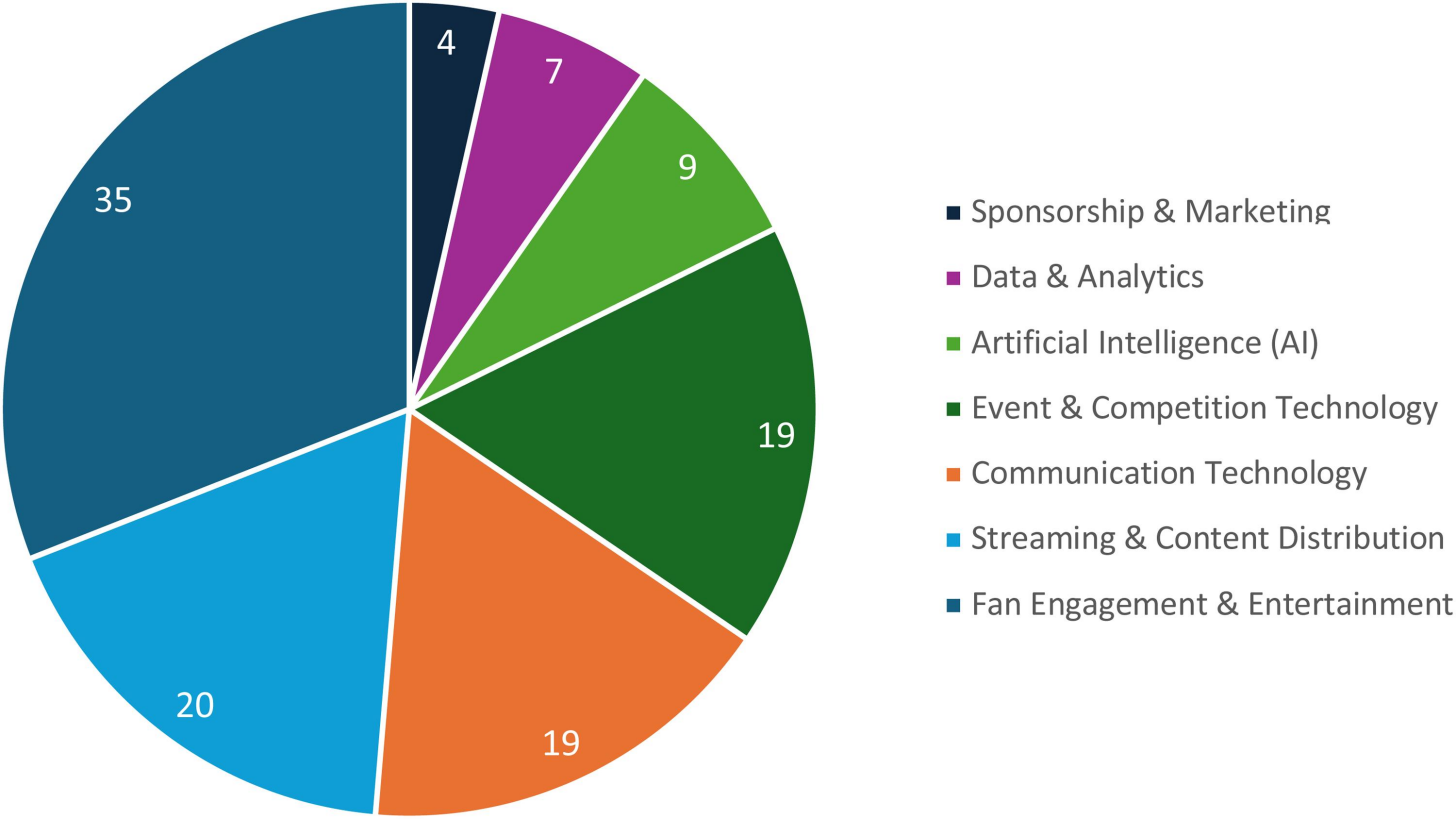
Proportion of each initiative's primary technology implemented by Summer IFs.

During our research process, we organised the initiatives into key technology classes for ease of summary and analysis. We have based myself on real categories used in the industry in general (20) and their purpose and core elements. They are the following, by alphabetical order: Artificial Intelligence, Communication Technology, Data & Analytics, Event & Competition Technology, Fan Engagement & Entertainment, Sponsorship & Marketing, and Streaming & Content Distribution. The categorisation enables a systematic review of how IFs integrate various technologies to enhance fan engagement and operations efficiency. Please note that most of them are classified by their purpose, except for the following category: Artificial Intelligence. We selected this category outside of communication or Data & Analytics as it is a growing technology in the sports environment (2). Therefore, our article wants to highlight its contribution on a separate level. Figure 6 shows the proportion of each initiative's primary technology implemented by Summer INternational Federations.

The pie chart above illustrates the distribution of technological initiatives grouped by primary technology category related to fan experience in the Summer Olympic International Federations. It is important to note that only the primary technology category is used to classify these initiatives.

The chart shows that the most significant proportion of initiatives (31%) falls under Fan Engagement & Entertainment, which reflects the considerable emphasis IFs place on technologies designed to enhance the fan experience. This category encompasses a broad range of initiatives aimed at creating immersive, interactive, and engaging fan experiences at events.

Then, the Streaming & Content Distribution category (18%) follows closely, indicating a strong focus on delivering content to fans across digital platforms, essential for increasing accessibility and engagement. In addition, Event & Competition Technology (17%) also plays a key role, highlighting efforts to improve the efficiency and appeal of events by integrating technology. Thus, it is particularly relevant in managing live sports events. Furthermore, real-time data is crucial in that organisation, ensuring seamless event delivery and engagement.

On the other hand, Communication Technology (17%) also emerges as an important category, reflecting how IFs leverage technology to communicate with fans, sponsors, and stakeholders. In contrast, Artificial Intelligence (AI) (8%) and Data & Analytics (6%) represent smaller segments of technological initiatives. Specifically, AI is used to personalise content and automate fan interactions, while data analytics helps IFs understand fan preferences and behaviours, facilitating more targeted engagement strategies.

Finally, Sponsorship & Marketing initiatives, although the smallest category (3%), still demonstrate the importance of using technology to enhance sponsorship opportunities. In addition, technology plays a role in delivering targeted marketing campaigns, helping IFs strengthen relationships with sponsors.

**Figure 7:**
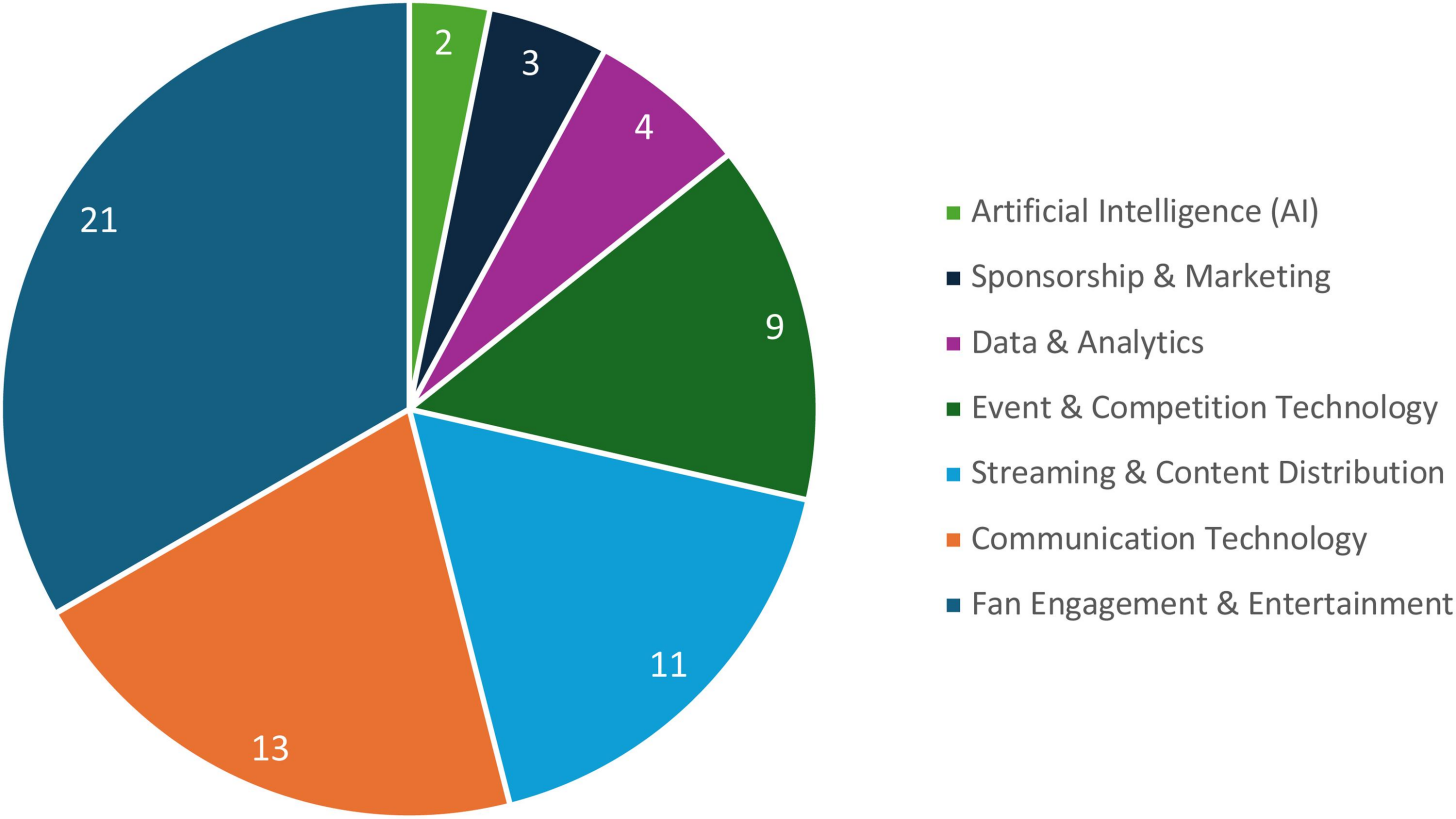
Proportion of each initiative's primary technology implemented by Winter IFs.

The distribution of initiatives across these categories shows that fan engagement remains the central focus of technological innovation within IFs. The significant investments in Fan Engagement & Entertainment, alongside the growing use of Streaming & Content Distribution and Event & Competition Technology, demonstrate IFs' dedication to improving the fan experience through digital and technological advancements. While AI and data analytics are still emerging areas, their potential to shape the future of fan engagement is noticeable. Figure 7 shows the proportion of each initiative's primary technology implemented by Winter International Federations.

In contrast, the pie chart above illustrates the distribution of technological initiatives grouped by category, all related to fan experience in Winter Olympic Federations. It is important to note that only the primary technology category is used to classify these initiatives.

As seen in the chart, Fan Engagement & Entertainment represents the most important proportion of initiatives (33%), indicating a strong focus on technologies designed to enhance the fan experience, thus making it a priority for Winter IFs.

Following this, Communication Technology (21%) is also a key area of focus. Thus, Winter IFs effectively leverage technology to engage with fans and stakeholders. Streaming & Content Distribution (18%) reflects significant investment in making content accessible to fans across digital platforms. Similarly, Event & Competition Technology (14%) focuses on improving the efficiency of event delivery, often incorporating real-time data and technology to enhance the fan experience during competitions.

Smaller categories include Data & Analytics (6%), which plays a crucial role in understanding fan preferences and behaviours, and Sponsorship & Marketing (5%), which reflects technology integration into marketing strategies. Artificial Intelligence (AI) accounts for the smallest proportion (3%), suggesting that AI could be used more for advanced fan engagement efforts.

From the distribution of initiatives, Winter IFs, like their summer counterparts, prioritise Fan Engagement & Entertainment. On the other hand, the investments in Streaming & Content distribution and Communication Technology reflect the motivation to improve fans' digital experiences and engagement across multiple touchpoints. While AI and Data & Analytics are still emerging, they are expected to play a more significant role in future fan experiences and engagement strategies.

In fact, as Winter IFs prepare for the Milano Cortina 2026 Winter Games, their digital transformation is accelerating. Moreover, on the same subject, research by N3XT Sports (11) shows that while 75% of Winter IFs collect fan data, they do so through more touchpoints (62.5%) compared to Summer IFs (50%). It also suggests a growing focus on digital engagement.

Furthermore, Winter IFs show a higher adoption of mobile apps (37.5%) and online fan-predictor games (37.5%) than Summer IFs. It indicates a shift towards more interactive and personalised fan experiences. In addition to this progress, Winter IFs can learn from the more digitally mature Summer IFs to continue to improve in this field.

#### 3.1.2 Technologies used on-site and remotely

The interviews showed explicit agreement between International Federations (IFs) of the growing importance of technology in improving the fan experience. Let me clarify here: when I did the interviews, I asked them about the technologies used at international sports events in general, and then, the technologies used at their international sports events. What I mean by that is the events directly organised by the IF. Panellists thus described various digital tools, ranging from data visualisation and mobile apps to streaming and interactive stadium experiences. Their answers revealed a double focus: to improve both the on-site experience for fans attending events and the off-site experience for viewers watching contests at home.

Several interviewees noted that data-based improvements are central to fan engagement. For example, Stéphane Schwander (FEI2) mentioned that “*a big trend is to use data and certain screen elements aligned with video streaming of the sports*”, allowing fans to personalise their viewing experience through overlays and customised dashboards. Similarly, Bernardo Franco (Volleyball World) noted that “*we are capturing more information, more statistics about the match* *…* *this gives these commentators more accurate information*” to improve broadcast coverage. Most federations also focus on live engagement through apps and websites. Harsimran Singh Virk (FIH1) explained how they “*worked on this front end of our website, where users can come and follow any international hockey match live*”, showing how they had moved from simple score reporting to more advanced, interactive features like head-to-head comparisons and live statistics. Ian Troseder (ISU) agreed, explaining how “*having an app or a web-friendly platform* *…* *enables fans to watch the event but then engage more directly with that event through a device*”.

Interviewees often mentioned venue-specific technologies such as big screens, live data displays, and fan interaction games when describing on-site events. Gaspard Dufour (FEI1) described new technologies to track athlete-horse combinations using camera-based technology, “*really committed to adding more information to the fans*”, and on-screen moving graphics and LED competition boards. Likewise, Bernardo Franco described using fan cams and interactive games during breaks, noting “*we can do something that they do in the NFL and the NBA like the kiss camera* *…* *so that the fans be engaged*”.

Interviewees also pointed out the importance of immersive technologies. Ekaterina Glebova highlighted that “*many technologies and technological tools* *…* *XR, digital twins, IoT, mobile apps, big data, and AI*” contribute to creating more interactive and personalised experiences.

Finally, the interviews also showed some of the problems federations face, such as balancing cost, infrastructure, and digital skills. Adrien De Cheveigné (UCI) declared that “*sports have always been one of the ways to develop new services*”. However, in sports such as cycling, giving a consistent experience over large, open areas remains a significant technical challenge.

#### 3.1.3 Immersive innovations: AR, VR and AI applications

The interviews revealed a shared curiosity and cautious optimism among International Federation representatives regarding the evolving role of Virtual Reality (VR), Augmented Reality (AR), and Artificial Intelligence (AI) in enhancing fan experience. Interviewees described these technologies as promising tools to improve interactivity and personalisation, but they also pointed out limitations related to accessibility, cost, and implementation feasibility.

AI emerged as the most widely adopted and discussed technology, particularly for its automation and content generation role. As Bernardo Franco (Volleyball World) explained, “*we have a supplier that does automated clipping* *…* *just after the match, the clip is ready*”. Similarly, Paul O'Neil (FIG) spoke about advanced uses of AI in content tagging and automatic writing: “*we just use a generative AI to write the article* *…* *it would do the article within seconds of the race finish*”. Harsimran Singh Virk (FIH1) echoed this, highlighting internal efficiencies: “*we run our enterprise solutions on Microsoft* *…* *Co-pilot is doing all the minutes of meetings*”. Several interviewees also explored the potential for AI in the future to further automate storytelling and engagement. However, many warned that AI still struggles with realistic visual representation, as Stéphane Schwander (FEI2) noted: “*you generate horses using AI, they sometimes have five legs* *…* *this is problematic*”.

Regarding Augmented Reality, several federations are already experimenting with commercial and broadcast use. As Bernardo Franco (Volleyball World) noted, “*we use AR to include that brand in your TV feed and then expose and sell that to the markets that you can*”. Similarly, Gaspard Dufour (FEI1) explained that AR is currently “*a lot about aesthetics* *…* *not as such providing more information*” but that it is useful for improving the visual quality of broadcasts. Ian Troseder (ISU) also noted its potential in advertising: “*we were able to offer augmented reality experiences that allow us to bring on different partners but still get the exposure*”. Several interviewees viewed AR as more applicable than VR, but they also emphasised its technical and financial limitations.

Interviewees discussed VR more cautiously. Most of them agreed that it has the potential to create immersive experiences, but many questioned its scalability and current value. For instance, Stéphane Schwander stated, “*I see VR more as a tool for developing educational or training projects rather than delivering fan engagement experiences*”. Adrien De Cheveigné (UCI) connected VR more to gamified contexts, such as “*cycling e-sports* *…* *where you connect your bike to a machine and you produce an effort that will reproduce whatever effort you're making with your avatar*”. Similarly, Ekaterina Glebova remarked, “*VR allows fans to experience games from unique angles* *…* *or even attend virtual stadium tours*”.

Several interviewees raised the issue of profitability and long-term sustainability. For example, Gaspard Dufour (FEI1) warned that “*those technologies actually have* *…* *high-cost implications* *…* *[and] the number of units where we can implement it remains limited*”. Similarly, Harsimran Singh Virk (FIH1) pointed out that “*they come with a lot of cost* *…* *we do not have that kind of big budget pockets yet*”. Adrien De Cheveigné (UCI) cautioned that while the technology is promising, “*you cannot place your money in the best position at that time*” if the market is not ready. The consensus was that only large-scale organisations or mega-events like the Olympics can afford widespread integration of these technologies.

### 3.2 Q2. How international sports federations use new technologies that could improve user experience at their sports events?

#### 3.2.1 Technology layering and innovation depth

**Figure 8:**
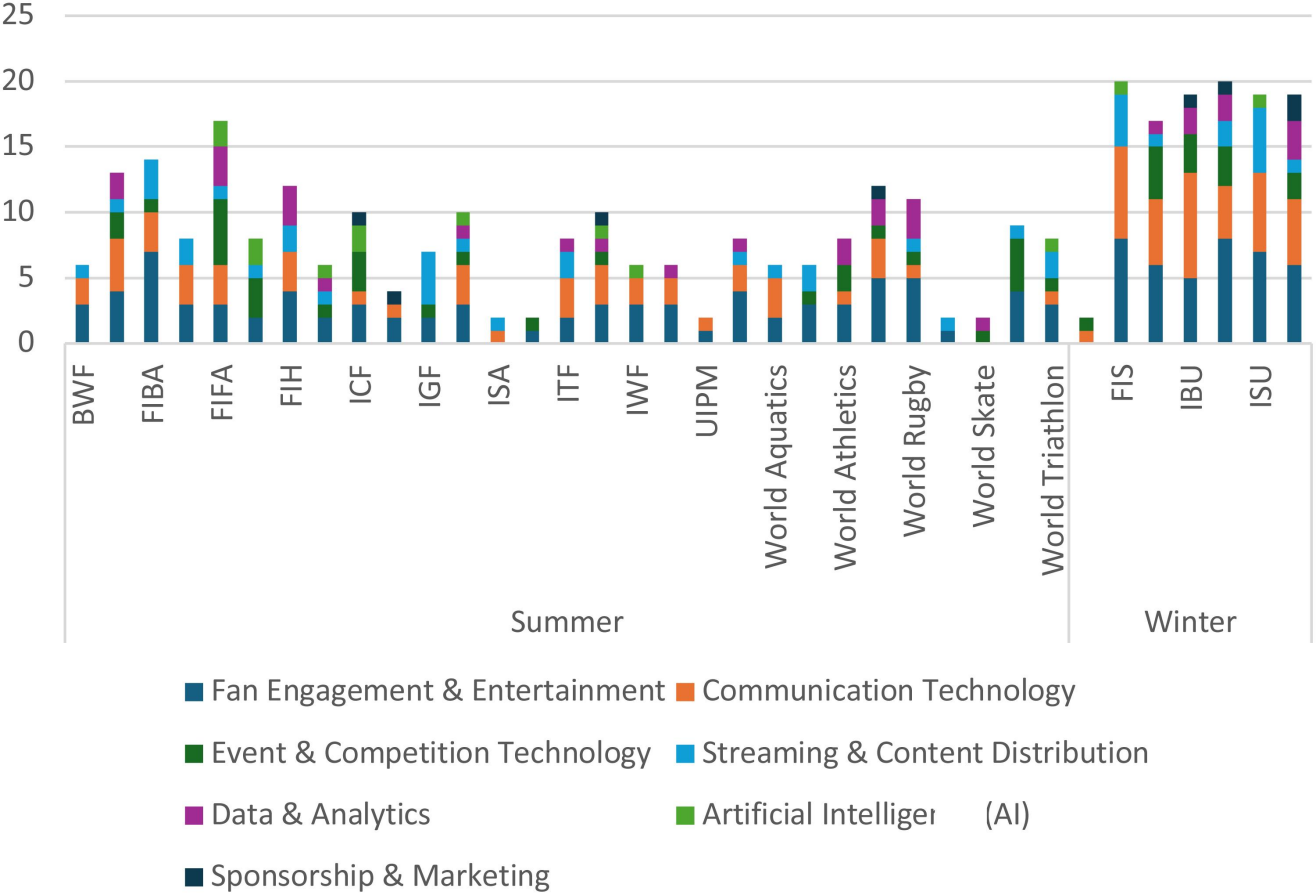
Representation of the addition of primary and secondary technologies in the Summer and Winter IFs initiatives.

Figure 8 is representing the addition of primary and secondary technologies in the Summer and Winter IFs initiatives. The figure above presents a stacked column chart illustrating the number and diversity of technological initiatives implemented by each International Federation (IF). On the *x*-axis, the chart lists each IF individually, with Summer IFs positioned on the left and Winter IFs on the right. Meanwhile, the *y*-axis indicates the total number of technology-related initiatives collected. Thus, the chart divides each column into segments that represent specific technology categories: Fan Engagement & Entertainment, Communication Technology, Event & Competition Technology, Streaming & Content Distribution, Data & Analytics, Artificial Intelligence, and Sponsorship & Marketing.

From this visualisation, a few key findings emerge. Firstly, even though Summer IFs collectively account for a higher number of total initiatives, Winter federations demonstrate a higher average number per federation. Some factors could explain this difference. One possibility is that Winter IFs simply implement more technological initiatives. Alternatively, these federations may be more transparent or proactive in publicly communicating their efforts. A final explanation relates to the research process. Since the data collection for Winter IFs happened after the Summer IFs, the increased experience with the methodology led to a deeper investigation. Moreover, the smaller number of Winter IFs might have allowed for more detailed exploration, potentially creating a bias in the depth of information collected. As a result, organisations such as FIS, IBU, IIHF, ISU, and WCF each implement more than 15 initiatives.

Secondly, Fan Engagement & Entertainment is the most frequently used category when comparing technology types. Many federations also adopt Communication Technology and Event & Competition Technology, which follow closely behind usage. In contrast, technologies such as Artificial Intelligence and Data & Analytics are relatively less frequent. For example, FIFA and FIG among Summer IFs and IBU or IIHF among Winter IFs present a more advanced integration of AI tools.

Moreover, the stacked format of the graph highlights the heterogeneity in technology use across federations. Furthermore, it shows that some IFs focus primarily on one, two or three categories, while others adopt a more balanced mix of technologies. It suggests that federations operate at different stages of digital maturity and may follow varied priorities depending on internal capacities, the board's strategy, or event types.

#### 3.2.2 Personalisation through fan data and CRM tools

Across the interviews, participants consistently acknowledged that using fan data to create personalised experiences is still a work in progress. While many international federations recognise its importance, they are at different stages of development. In several cases, interviewees admitted that their organisations are only beginning to explore how to collect and use such data effectively.

To begin with, Gaspard Dufour (FEI1) explained that although the federation has collected a large amount of data on horses and athletes, they are not yet using it to personalise the fan experience. He stated, “*We have all those good ideas on paper* *…* *but in practice, the way it's implemented is very few*”. He also noted that the federation would like to connect fans with their favourite discipline or athlete but does not have the right systems in place to do so.

Similarly, Stéphane Schwander (FEI2) described plans to build a Customer Data Platform (CDP) to combine data from different sources and help automate personalised communication. However, he clarified, “*Currently, we only understand fan data through native analytics from platforms like social media or Google Analytics*”. Therefore, much of the potential for personalisation remains untapped. Likewise, Bernardo Franco (Volleyball World) noted that personalisation is limited to the customer relationship management (CRM) system and the OTT platform, where fans can select their language and favourite leagues. He added, “*At the moment, I don't feel that we have developed our solutions to get to this level of customisation*”. He also shared the ambition of letting fans choose their camera angles during matches, similar to the experience offered in Formula One. However, he said this is not yet possible due to technical limitations. Moreover, Adrien De Cheveigné (UCI) highlighted that UCI uses data from its fan engagement programme, My World of Cycling, to understand audience preferences better. Based on user interactions, they try to adapt content and messaging. However, he confirmed, “*We are at the very beginning of that*”. He also pointed out that the lack of ticketing data and the limited volume of fans make it difficult to develop fully tailored services.

Despite these challenges, some federations have started using specific tools that support basic levels of personalisation. For example, Adrien De Cheveigné (UCI) mentioned Azuu, a design platform that helps create graphics adapted to specific fan groups, and Grabyo, a live video editing tool. Similarly, Ian Troseder (ISU) outlined future goals to offer personalised websites and content based on user activity. He explained, “*We will know that* *…* *these are the things that this person has looked at, so therefore we'll have their own personalised experience*”. In addition, Harsimran Singh Virk (FIH1) emphasised the value of mobile apps that could show fans content linked to their favourite team or player. He imagined a platform where users could receive updates about matches, statistics, and videos specific to their interests. He also noted that such technologies would be valuable if integrated with their OTT service, though budget remains a barrier.

For on-site engagement, Cyril Nivel (ICF) shared ideas for future tools like prediction games or judge-style apps, allowing fans to interact based on their expertise or location. He also explained how the federation used a chatbot with different difficulty levels at the Paris 2024 Games to engage fans, from casual viewers to dedicated followers. However, he admitted, “*We are starting to get a better view of our fans, but I don't think we have strong data analysis yet*”.

Paul O'Neil (FIG) confirmed a similar situation. He stated that although they have a vision to use first-party data, they have not yet taken the first steps. He recalled advice from a UEFA colleague who said that even a simple email address could be the starting point to build more personalised services. Nevertheless, Mr. O'Neil (FIG) observed that internal adoption of such ideas remains challenging due to limited resources and the need to demonstrate the benefits.

Finally, Ekaterina Glebova pointed out that while most federations are still exploring these options, technologies like big data and AI already play a leading role in personalisation elsewhere. She cited UEFA EURO 2024 as a good example, where fans receive minute-by-minute updates through personalised feeds.

#### 3.2.3 Monitoring fan behaviour and adapting to preferences

Interviewees from several international federations explained how they track fan behaviour and use this information to offer more personalised experiences. While most recognised the value of collecting fan data, many also admitted they are still at an early stage of using it effectively. To start with, Bernardo Franco (Volleyball World) mentioned that video analytics are a key tool for understanding what fans watch. He explained: “*We do have all of the video analytics* *…* *how many plays were started, what is the average viewership, or how long do the fans keep watching the selection of videos we have*”. This data helps them compare different leagues, such as the Beach Volleyball and Nations League, and improve their content strategy. He also explained that the federation mainly uses ticketing data to evaluate the success of marketing campaigns rather than to measure fan engagement directly. Likewise, Stéphane Schwander (FEI2) shared that they look at digital interactions on their website and social media. He said: “*For digital touchpoints like the website, we track unique visitors and the average time spent on pages*”.

Regarding social media, his most important indicator is engagement: “*Engagement is the most important KPI because it shows intentional interaction. Reach alone doesn’t mean much*”. Similarly, Ian Troseder (ISU) described how they study fan behaviour using their streaming platform and website. He explained: “*Some of the key metrics* *…* *are what did they watch and for how long did they watch it, and if they stopped watching, why?"*.  He also said they monitor website visits and look at where users stop reading to improve content: “*Which kinds of content are they interested in, how often, how long do they stay* *…* *and if they leave, where do they leave?"*.

Cyril Nivel (ICF) added that they can already track valid data through YouTube: “*We can track how many minutes people watch, which discipline they follow, which pages they visit, and from which country*”.  He also shared that their team created a chatbot for the Games, with different levels for different audiences (Gold, Silver, and Bronze), to help manage fan engagement more easily. On his side, Paul O'Neil (FIG) said that while they do not yet offer personalised experiences, they do rely on social media and website analytics to understand their audience. He also mentioned a recent research project that helped them see apparent differences between fan groups: “*We did a big audience research project* *…* *that helped us to understand whether the audience is the same for all the different disciplines*”.

Finally, Ekaterina Glebova listed four key types of fan behaviour that can help international federations offer more personal experiences:

- Social media interactions
- Digital platform engagement
- Attendance
- Purchase behavior patterns”

#### 3.2.4 Data integration and centralisation in IFs

Interviewees highlighted a wide range of practices in how International Federations (IFs) manage fan data across different platforms. Some federations are in the early stages. Gaspard Dufour (FEI1) explained, “*Right now, very simple. We just use our CMS system […] to capture data*”, mainly email addresses and basic demographics, with “*no automation*” and “*manual exports*” used for newsletters. Paul O'Neil (FIG) similarly noted, “*Other than social media, no*”, adding, “*We would like [integration] this year, ideally. I think we're behind other federations*”.

Others have made significant progress. Bernardo Franco (Volleyball World) stated, “*We do have a central place in Snowflake that brings together all of the fan data from any potential source*”, including OTT, ticket sales, websites, and national federations. Ian Troseder (ISU) noted the use of a data warehouse developed with Two Circles and integrated through Zoomph: “*We collate it in a platform […] called Zoomph”,* which also includes streaming and TV data from sources like Google Analytics and Nielsen.

Harsimran Singh Virk (FIH1) explained that fans register via their website and data is managed through Zoho Campaigns: “*We are using Zoho campaigns […] we store the email addresses and […] send them content*”. Similarly, Adrien De Cheveigné (UCI) described data collection via “My World of Cycling” and integration into media or newsletter systems using APIs. He noted that integration happens “*mostly whenever we can access it*”, and must comply with GDPR and legal constraints.

Some federations acknowledged a lack of systematisation. Stéphane Schwander (FEI2) described a limited and largely manual integration process, using SQL databases: “*We don't have consolidation, we don't have centralisation of the different data sources*”. Cyril Nivel (ICF) echoed this: “*We are not managing it fully yet […] We want to do it*”, illustrating early-stage digital development. Ian Troseder added, “*There isn't one platform which does it all, so it's just a case of bringing it and pulling it into reports*”, relying on external consultants for data consolidation.

Despite challenges, centralising fan data is widely recognised as vital to enhancing fan engagement. Gaspard Dufour (FEI1) stated, “*Yes, I believe it*”, explaining that consolidation helps identify fan types — from athletes to organisers — and build narratives around them: “*If you consolidate data, we can create those stories. If you do not, you just have no clue*”. Bernardo Franco (Volleyball World) confirmed: “*Absolutely*”, noting that centralisation enables organisations to understand fan interests and purchasing behaviour. Stéphane Schwander (FEI2) similarly affirmed, “*Yes, I believe so*”, highlighting the benefit of understanding core audiences.

Some respondents took a more measured view. Harsimran Singh Virk (FIH1) admitted, “*I don't know about better fan engagement or not, but it certainly helps us to know our fans better*”. Adrien De Cheveigné (UCI) said, “*I think in the long run it will, but it definitely takes some time to build*”, underlining the need to align with fan expectations. Ian Troseder (ISU) provided a concrete example: “*We were able to use that database, segment it […] and we sold a few more tickets*”.

Cyril Nivel (ICF) stressed the importance of nuance: “*We can improve the fan engagement by defining some slots. It was interesting and targeted*”. Paul O'Neil (FIG) referred to UEFA's example: “*Until you have that, you can't do anything*”, linking centralised data with commercial success and product strength. Ekaterina Glebova concluded, “*Yes, since it helps to sort and operate data”, "confirming its operational value and strategic importance*”.

#### 3.2.5 Digital maturity

Most interviewees acknowledged the critical role of digital maturity in their federation's strategic development, though the degree of advancement and resourcing varied considerably. While some federations have embedded digital transformation as a central pillar of their strategy, others remain at early stages of development, often constrained by limited budgets or legacy systems.

Gaspard Dufour (FEI1) highlighted the importance of digital tools to senior leadership: “*Digital maturity, overall strategy, technology, and digital status—I think, very important. We can just answer that because it has always been very much supported by the top management*”. He distinguished between strong digital maturity in sport administration vs. the fan engagement side, noting: “*The maturity and the pure digital side linked to fans is still to be improved*”. Bernardo Franco (Volleyball World) similarly affirmed the relevance of digital maturity, stating: “*It's important as it's important for any entertainment actor today*”. However, he also acknowledged practical limitations: “*We do not have the resourcing to tackle this as an absolute priority internally*”. He noted that resource constraints remain challenging while the federation has accumulated experience through various projects, such as its website, OTT, and app integrations.

Stéphane Schwander (FEI2) reinforced this idea, linking maturity to leadership support: “*Digital maturity comes from the understanding of it by top management. Thus, it sets the culture embraced by the FEI*”. However, he expressed concern over the pace of transformation: “*The world is accelerating far more quickly than how the FEI is embracing new technology and digital transformation*”.

Other respondents, such as Jon Wyatt (FIH3), underscored how digital is now inseparable from overall strategy: “*We don't even talk about digital strategy because everything is digital, right? It just has to be*”. He stressed that digital channels enable broader reach: “*I might have 50 kids down there, but if we do a really good piece of engaging content online, we could get 50,000*”. Adrien De Cheveigné (UCI) also viewed digital maturity as “*highly important*” and described the need to adapt strategies based on disparities across national federations and different fan communities. He cited advanced efforts like the UCI Track Champions League and partnerships with Warner Bros. and Amazon Web Services as examples of digital experimentation. He noted, however, that “*you also need to educate your fans on those services*” and manage expectations carefully based on context and available resources.

Ian Troseder (ISU) openly described the organisation's current digital state as “immature” and pointed to new leadership support as a positive sign: “*From a head office point of view, people understand where we need to go to with digital*”. He listed key next steps, including developing an in-house streaming platform, expanding into Chinese social media, and eventually launching a global ticketing system to “*take more control and also drive revenues*”.

On his side, Cyril Nivel (ICF) emphasised the strategic necessity of digital development: “*If you are not in this world, you are nothing*”. He acknowledged that “*we are not at this* *…* *we are never [finished] because digital is evolving very well*”, pointing to continuous adaptation and upcoming team expansion as steps forward: “*The team is still two. There will be ten soon*”.

Otherwise, Paul O'Neil (FIG) was frank about his federation's digital lag: “*We're not digital mature. So that's the first step—we need to get there*”. He shared that the organisation had used “*Microsoft Access*” a system no longer supported until recently. He advocated for complete platform replacement: “*Let's just put them on a new platform where we can really use them properly*”. For Paul O'Neil, success hinges on “*finding the right partners*”, centralising data systems, and building from first-party data toward broader personalisation strategies.

Finally, Ekaterina Glebova summarised the general sentiment: “*It seems to be important, and there is room for improvement in most federations nowadays"*. She outlined a six-step roadmap toward maturity: “*1. Digital audit, 2. Strategy building, 3. Digital and traditional infrastructure adjustment, 4. Personnel training, 5. Stakeholder engagement, 6. Continuous improvement*”.

#### 3.2.6 Standout practices and emerging success models

In analysing the responses from various federation representatives regarding best practices in enhancing fan experience through technology, several innovative approaches emerged:

##### 3.2.6.1 Maximizing the use of LED displays

Gaspard Dufour (FEI1) highlights the strategic use of LED technology within arenas to enrich the spectator experience: “*We did a big step, and we put a lot of animated content on LEDs. So not only on big screens but on all those LEDs* *…* *Presenting the obstacles on the board, being so interactive with dynamically changing the board and those kinds of things, onboarding the scores, etc*”. This approach leverages dynamic visual content to provide insightful information previously inaccessible to in-person audiences, thereby deepening their engagement with the event.

##### 3.2.6.2 Automated content creation using artificial intelligence

Bernardo Franco (Volleyball World) discusses implementing AI-driven tools to automate the generation of personalised video clips: “*We do have a supplier that does automated clipping. So, they receive all our videos from our beach volleyball and indoor volleyball events* *…* *After the match, this clip is automatically created, and we can set workflows so most of this video is automatically posted or sent somewhere*”. By providing players, teams, and sponsors with immediate access to tailored highlights, this practice enhances fan engagement through timely and relevant content sharing.

##### 3.2.6.3 Branded content creation as a media platform

Stéphane Schwander (FEI2) introduces the concept of federations acting as media platforms to produce content in collaboration with brands: “*Creating content—both branded and organic—for fans, for brands, or in partnership with brands is key. This triangular model, where a brand funds content creation by the FEI, allows us to craft content that serves the fans while aligning with brand values*”. This strategy diversifies content offerings and ensures authenticity, resonating with audiences and fostering deeper connections.

##### 3.2.6.4 Development of comprehensive data hubs

Harsimran Singh Virk (FIH1) emphasises the creation of accessible data repositories for fans: “*We've created a data hub that requires a user login. This hub contains data on all teams and players* …  *We've tried to make this data more visually engaging and accessible to fans*”. This initiative uses advanced tools like Power BI to democratise data access, allowing fans to engage more deeply with statistical aspects of the sport.

##### 3.2.6.5 Adapting fan engagement to unique event environments

Adrien De Cheveigné (UCI) discusses tailoring fan engagement strategies to the distinctive settings of cycling events: “*We are highly dependent on the weather, we are highly dependent on the available internet connection*  … *So we need to make sure that we are heavily reliable whenever we try to deliver that fan experience*”. Thus, by focusing on reliability and accessibility, even in outdoor environments, this approach ensures constant and attractive fan experiences.

##### 3.2.6.6 Emphasising emotional connections in fan experiences

Cyril Nivel (ICF) underscores the importance of capturing emotional moments to enhance fan engagement: “*Fan experience is about emotion. We work with experts who can create and share that emotion* … *Some of the best moments of the Games are those emotional connections*”. This perspective highlights the value of storytelling and emotional resonance in creating memorable fan experiences.

## 4 Discussion

Through a discussion, we can now will argue, reflect, and bring theory to the objective of validating or noting the hypotheses built through the literature's review. This section builds on the results and theoretical framework developed throughout the research. It also integrates new academic and professional sources to clarify the key analytical themes and support the observations made. In other words, the discussion uses theory to interpret the findings presented in the results section constructively.

In fact, the next section of our article should be approached with considerable caution and humility. Indeed, while we have attempted to formulate hypotheses based on our review of the literature on the subject, we cannot, however, consider our methodology sufficiently robust to be generalizable. It seems more reasonable to consider it as illustrative of observed phenomena. The size of our sample is also insufficient, given our ambition. We will address these points again later in the article.

### 4.1 Discussion on hypothesis 1

*H1: Virtual Reality (VR), Augmented Reality (AR), and Artificial Intelligence (AI) are the most frequently used technologies to enhance the fan experience at international sports events, and their implementation is profitable and sustainable in the long term.*

The results show that immersive technologies are being adopted unevenly across International Federations (IFs). They adopt VR, AR, and AI at different levels, even though all three technologies can potentially improve the fan experience.

#### 4.1.1 Uneven adoption of VR, AR, and AI

First, only a few federations have implemented VR, which remains in its early stages. For instance, the Golf Federation, Sailing Federation, Ice Hockey Federation, and Table Tennis Federation have developed immersive features such as 360-degree viewing, VR games, and enhanced replays. However, these initiatives remain niche (e.g., targeting limited audiences or specific events). Many federations have not adopted VR more broadly due to high costs, complex technical requirements, and limited accessibility for fans.

Compared to VR, AR is slightly more advanced and has seen more applications. Sports such as sailing, skiing and motorsports use Augmented Reality in visual presentations and advertising. These implementations range from AR overlays to sponsor integration and on-screen data enhancements. Despite growing interest, this limited uptake shows that this technology has not yet become a standard feature across federations. Although many acknowledge AR's potential to enrich the fan experience, cost, complexity, and technical barriers hinder broader implementation.

#### 4.1.2 AI leads the way, but traditional tools still dominate

Artificial Intelligence stands out as the most widely used of the three technologies. According to PwC (2), 50% of IFs have already implemented Generative AI (GenAI). The study's findings confirm that several IFs, including WTT, the FIG, the FEI, FIA, and FIFA, use it for content creation, video automation, judging systems and performance analysis.

Moreover, the 2024 Sports Technology Annual Review by The STA Group highlights two key impacts of GenAI: First, the automation of content creation, significantly reducing time and manual input. The review notes, “with GenAI and LLMs, and its ability to create text that sounds natural, content that once took hours to produce can be automated and published in near-real time with minimal to no manual revisions”. Second, it warns that fans still demand high-quality, authentic content. As stated, “sports fans are not so forgiving when it comes to the content they consume … extra care is needed when considering serving GenAI-based content, to maintain a high-level overall experience that looks genuine and doesn't compromise on audio, video, captions, etc.”. Finally, many federations view AI as more affordable and easier to implement than AR and VR.

Nevertheless, beyond immersive tools, most federations continue to rely on more established digital technologies. These include websites, mobile apps, and social media platforms. Federations also use data visualisation tools and on-site features like live stats, big screens, and interactive games. These solutions are more accessible, require fewer resources, and still provide added value to fans. The initiatives' analysis reflects this trend: the Fan Engagement & Entertainment category holds the largest share, representing 31% of Summer IFs and 33% of Winter IFs' initiatives.

### 4.2 Discussion on hypothesis 2

*H2: Artificial Intelligence (AI) and Customer Relationship Management (CRM) systems enhance significantly fan experience through personalisation.*

The analysis of interviews and initiatives suggests that many International Federations are increasingly exploring Artificial Intelligence and Customer Relationship Management systems to improve the fan experience, primarily through personalisation. However, most federations are still in early development stages and face several challenges in implementing these technologies effectively.

#### 4.2.1 CRM systems: opportunities and limitations

Some federations have taken concrete steps to use CRM systems. For example, Volleyball World combines its CRM with an OTT platform, enabling fans to select languages and favourite leagues. The UCI uses the “My World of Cycling” platform to collect data and tailor content based on user preferences. The ISU has plans to introduce personalised websites that adapt content according to fan behaviour. Meanwhile, UWW and the IBU have implemented CRM tools to improve organisational communication.

Despite these promising cases, most IFs have not used CRM for complete personalisation. Interviewees from federations such as the FEI and the FIG acknowledged that although they collect data from fans and athletes, they currently lack the systems or internal capabilities to use this data effectively. Due to limited digital infrastructure, some federations also struggle to connect fans with specific disciplines or athletes.

Furthermore, the analysis of technological initiatives rarely mentions CRM systems. Only one Summer IF (UWW) and one Winter IF (IBU) referenced CRM usage explicitly. More federations may use CRM tools without publicly communicating these efforts, especially if these systems support internal operations more than visible fan engagement.

#### 4.2.2 Artificial intelligence for personalisation and content

The 2025 *Digital Trends in the Sports Industry* report by N3XT Sports (21) highlights the role of AI: “AI is transforming how sport properties personalize the fan experience by optimizing data-driven decision-making”. It emphasises how sport organisations can use AI to enhance the fan experience by offering tailored experiences based on user data.

A few IFs have already begun integrating AI into specific functions. The FIVB uses AI-powered tools from WSC Sports to create automatic highlight videos. FIG and ISU are experimenting with AI to improve judging systems, aiming to boost fairness and transparency. These factors also impact the fan experience. FIFA has implemented semi-automated offside technology to help explain referee decisions to fans. Likewise, the FIS is testing AI tools to personalise automatically video content for social media.

However, AI is used more for content production and enrichment than personalising the fan journey. As Zok (22) observes, “sports organisations that integrate AI into their operations reduce inefficiency by a rate of at least 50 per cent and develop new value for all stakeholders”. Thus, this observation can comfort federations' choices mentioned earlier that have implemented AI in their processes.

### 4.3 Discussion on hypothesis 3

*H3: International Federations that consolidate fan data from various sources achieve higher digital maturity and improve fan engagement than those with fragmented data systems.*

#### 4.3.1 Centralised data systems enable stronger fan engagement

Findings from both the qualitative interviews and descriptive data analysis support Hypothesis 3. The initiatives and interviews examined show a clear trend: Federations that aggregate fan data from multiple sources are more digitally developed and ready to engage with fans.

Sports federations implementing integrated systems are the first to adopt modern data tools and platforms such as *Snowflake*, *Zoomph*, or *Zoho*. For example, Volleyball World, the ISU, the UCI, and the FIH have embraced comprehensive data platforms that collect fan information from various sources such as streaming services, OTT platforms, ticket sales and websites. This integration enables them to segment audiences, tailor communications, and improve outcomes such as ticket sales, as in ISU's case. Additionally, these federations report higher levels of digital strategy, innovation and collaboration, all indicators of advanced digital maturity.

In contrast, federations like the FEI and FIG remain at an early stage. They acknowledge that they have not yet centralised their fan data. As a result, they cannot personalise content or familiarise themselves with fans' preferences. Still, interviewees unanimously agree that consolidating fan data is vital for personalisation, engagement, and strategic planning. Some participants confirmed that consolidating data at a central point helps federations understand who their fans are, what they like, and how to engage with them more effectively. It also makes storytelling easier, as well as targeted marketing and collaborations. One of the participants summarised: “If you centralise data, we can tell those stories. If you don't, you just have no clue”.

#### 4.3.2 Digital maturity and ROI depend on integration

Besides, federations with centralised data systems are best able to measure the ROI of their digital campaigns and tools. They measure performance based on metrics of engagement (e.g., social media, video views, and engagement metrics) or income (e.g., more subscriptions or sales). Thus, federations' systems of fragmented data struggle with measuring ROI or proving investments.

Finally, data integration also appears to go hand in hand with digital maturity. Federations with established platforms are likely to be more developed in general terms of their digital strategy. They are approved by senior management and use digital tools to stay competitive. One interviewee said, “We don't even talk about digital strategy anymore, because everything is digital”.

The visual analysis in the figure illustrating the number of primary and secondary technologies in Summer and Winter IF initiatives also support Hypothesis 3. The figure clearly shows that Winter IFs, although fewer in number, implement a higher average of technology initiatives per federation. Notably, federations such as the ISU, IBU, and IIHF demonstrate a broad adoption of tools across multiple technology categories. It has Data & Analytics and Artificial Intelligence, which are key indicators of data-driven strategies. In contrast, several Summer IFs show lower initiative counts and fewer categories covered. It indicates more basic digital systems. Finally, these findings align with interview insights.

Ultimately, these analyses align well with the “Clockwork” model developed by Glebova, Desbordes and Czegledi (23), underlining that effective technology implementation in the sports ecosystem depends on developing the proper infrastructure, services and innovation. This model highlights how digital infrastructures and data systems form the foundation for technological advancement in sport. In addition, the model confirms that without an operational base, including data consolidation, technology-based engagement programmes are unlikely to succeed. Therefore, federations that centralise fan data take a key step toward achieving digital maturity and enabling them to launch personalised initiatives (23).

## 5 Conclusion

“69% of fans report that the use of emerging technologies has enhanced their viewing experience, both inside and outside the stadium” (12).

This research explored how International Federations (IFs) use new technologies to improve the fan experience at their sports events. The study provided a partial overview of the digital tools and strategies currently used by IFs recognised by the International Olympic Committee (IOC).

The study is based on descriptive analysis of some initiatives by IFs related to technology and qualitative interviews with federation representatives were conducted. In this manner, by remaining modest and cautious, given the methodology we used, the results confirm that most federations are experimenting with different technologies to improve the spectator experience on-site and remotely. The results also show that the adoption of technologies varies depending on available resources, strategic vision, and digital maturity.

By developing, describing and trying to answer the research questions, the article study confirmed that federations with centralised data systems and a strong digital strategy are more likely to personalise fan experiences, make better use of digital platforms, and evaluate the return on their digital investments. At the same time, the study shows that even federations with limited budgets can still deliver engaging experiences using accessible tools such as mobile apps, social media, or live graphics.

Although this exploratory research was conducted in Lausanne (Switzerland), the heart of international federations, its focus on international governing bodies gives it global relevance. The findings also confirm insights previously found in grey literature, such as the importance of fan personalisation and the centralisation of fan data. Moreover, the findings underline the value of centralising fan data to improve personalisation, the importance of testing immersive tools before large-scale implementation, and the benefits of aligning digital strategies with fan expectations.

In addition, the sport ecosystem is already moving in the same direction. The International Olympic Committee, together with Deloitte, recently launched the Olympic AI Agenda to guide the future use of artificial intelligence across five key areas, including digital fan experience. Their goal is not only to improve operational efficiency but also to create more personalised, engaging, and ethical interactions between fans and the Olympic Movement (10). This initiative shows that the future of fan engagement depends on human-centred digital innovation. These global strategies highlight the need for all IFs to take part in this technological transformation.

The study shows that digital transformation in IFs is already underway, though it moves at different speeds across organisations. As one participant stated, international sports federations compete with the entertainment industry today. This reality makes digital agility more important than ever for the future of sport.

For several years now, the IOC has been considering how to modernize “its” Olympic Games in order to attract a younger audience and generate steadily increasing revenue. Many of the sports added since the 1990s seem to have been inspired by the X Games, which were created by the ESPN television network. BMX, surfing, slopestyle, halfpipe, boardercross, and skateboarding at the 2024 Paris Games have thus become almost “commonplace” in the eyes of the general public.

But the focus for the coming years is clearly on “electronic games”, not limited to esports or video games. Virtual and augmented reality should also help modernize the media coverage of sporting events. What form will this take? For now, no one knows. An “Electronic Olympics”? The inclusion of esports in the Summer Games? The creation of a parallel event managed by the IOC in the form of a franchise? A “hybrid” integration of technology that allows for a blend of traditional sports and at-home participation via a platform? This could be possible, for example, in cycling or running, and would give everyday athletes the chance to compete against the stars of their discipline.

There is therefore a real marketing challenge for sports federations in adopting this technology. While they are the ones who set the rules, for most of them, the Olympic brand is essential, and the Olympic Games provide a significant portion of their visibility.

## 6 Future research

The aim of our article was to bridge the gap between grey literature and consultancy reports.

While remaining humble, we believe we have partially fulfilled our initial ambitions, but it seems logical and useful to expand this research, which can be considered exploratory.

If we wish to further the research topic we have attempted to explore, it would be useful to develop an ambitious research program in the future.

From a methodological standpoint, our article has a number of limitations that could be addressed by adopting a more rigorous approach and design.

First, a thorough analysis must be conducted after rigorously formulating the hypotheses.

A more conclusive demonstration can only be achieved by using a larger sample of federations that is more representative of the highly heterogeneous nature of these organizations. Several typologies could be employed (federations with highly professional athletes vs. “amateur athletes”; long-established federation vs. newly created; “highly legitimate” federation vs. “highly threatened by the sports business sector”; individual sports federation vs. team sports; etc.).

Within each of these federations, the case study methodology (19) could be used, which would involve interviewing at least four different stakeholders holding key managerial positions within the organization. Within each federation, the process should then be replicated with individuals holding the same position. Ideally, six cases should be studied. If these interviews are supplemented with a few additional interviews with consultants/experts in the field and in technology, this would result in a minimum of 25–30 interviews.

A new topic that should be considered: consumer and fan experience.

As mentioned earlier, the study measures organisational intentions and practices rather than actual fan experience outcomes (satisfaction, engagement metrics, or behaviour outcomes).

Also, the causal link between data consolidation and improved fan engagement remains assumed rather than measured.

Stronger integration of concepts such as sport experience design, engagement, and digital maturity with established frameworks (e.g., service-dominant logic, customer experience theory, or digital capability maturity models) would improve theoretical contribution.

Therefore, if we are to address this issue properly, it is obviously necessary to draw on the extensive literature in marketing and consumer behavior, paying particular attention to the connections with technology (23–26).

Ultimately, to properly address the research question regarding consumers, their perceptions, and the effectiveness of the technology used, it seems essential to conduct a quantitative study among fans, spectators, and television viewers. In this regard, an interesting comparison with consumers of American professional leagues would be relevant, since we know that when a league is closed, its sporting appeal diminishes, and it is necessary to increase the use of entertainment to boost customer interest. In this regard, technology is undoubtedly one of the most fruitful tools for the years to come.

Here are the key elements that will enable us to conduct more relevant and comprehensive research, so that the exploratory findings we have identified can be confirmed or refuted.

## Data Availability

The original contributions presented in the study are included in the article/Supplementary Material, further inquiries can be directed to the corresponding author.

## Footnotes

^1^This article is based on a master's thesis (Rywalski, 2025) (27) written by Thomas Rywalski under the supervision of Michel Desbordes, who also contributed to the editing of the article.
